# Host specificity of Asian parasitoids for potential classical biological control of *Drosophila suzukii*

**DOI:** 10.1007/s10340-018-1003-z

**Published:** 2018-06-18

**Authors:** Pierre Girod, Océane Lierhmann, Teddy Urvois, Ted C. J. Turlings, Marc Kenis, Tim Haye

**Affiliations:** 1 CABI, Delémont, Switzerland; 2 Laboratory of Fundamental and Applied Research in Chemical Ecology (FARCE), Faculté des Sciences, University of Neuchâtel, Neuchâtel, Switzerland

**Keywords:** Spotted wing drosophila, Non-target effects, Host range, Fruit flies, Larval parasitoids

## Abstract

The Asian spotted wing drosophila, *Drosophila suzukii,* has recently become a serious pest of soft fruits in Europe. Classical biological control through the introduction of larval parasitoids from its native range in Asia is presently being considered. However, host specificity of potential biological control agents has to be determined to avoid releasing species that may have unintended non-target impacts. Larvae of six different European non-target fly species and the target *D. suzukii* were exposed either on diet or blueberries to three Asian larval parasitoids, *Asobara japonica*, *Leptopilina japonica,* and *Ganaspis* cf. *brasiliensis*, and one European species, *Leptopilina heterotoma*. *Asobara japonica* showed the lowest specificity, attacking and developing in all Drosophilidae. *Leptopilina japonica* successfully parasitized two non-target Drosophilidae, *D. melanogaster* and *D. subobscura*, with one singly progeny emerging from *D. immigrans*. *Ganaspis* cf. *brasiliensis* had the highest level of specificity but variations occurred between two geographical populations tested. A Japanese population was strictly specific to *D. suzukii,* whereas another population from China parasitized *D. suzukii*, *D. melanogaster* and sporadically *D. subobscura*. The European *L. heterotoma* successfully developed in *D. melanogaster*, *D. subobscura* and occasionally in *D. immigrans*, but nearly all eggs and larvae in *D. suzukii* were encapsulated. These results show that *Ganaspis* cf. *brasiliensis* is the species with the highest potential for biological control, but more studies are needed on its taxonomic status and the existence of biotypes or cryptic species varying in their specificity before field releases can be conducted in Europe.

**Electronic supplementary material:**

The online version of this article (10.1007/s10340-018-1003-z) contains supplementary material, which is available to authorized users.

## Key message

- *Drosophila suzukii* is a serious pest of soft fruit crops worldwide and classical biological control may provide an alternative to the use of chemical insecticides.
- Host specificity tests were conducted with three Asian and one European parasitoid species, using five European *Drosophila* spp. and one Tephritidae species as hosts.
- A population of *Ganaspis* cf. *brasiliensis* from Tokyo (Japan) showed the highest degree of host specificity and is the best candidate for classical biological control.

## Introduction

Globalization and climate change speed up the spread of new invasive pests, causing an estimated agricultural loss of more than $1.4 trillion per year worldwide (Pimentel et al. 2001). Since the second half of the twentieth century, around 30% of alien arthropod species had established in Europe have originated from Asia (Roques et al. 2009). Among these, *Drosophila suzukii* Matsumura (Diptera, Drosophilidae), or spotted wing drosophila. This fly of East Asian origin was first found in 2008 in Europe and North America and, in just a few years, it invaded several continents (Asplen et al. 2015; Fraimout et al. 2017) and became a very serious pest of many fruit crops worldwide as non-crop or ornamental fruits (Walsh et al. 2011; Lee et al. 2015; Kenis et al. 2016).

Current control methods rely on chemical insecticides or expensive and labour-intensive cultural practices (Haye et al. 2016). An alternative approach to control *D. suzukii* could be to use classical biological control, i.e. introducing natural enemies from the native range of the pest. Recent studies indicated that natural enemies from Asia may provide a better area-wide control of *D. suzukii’*s populations in the invaded areas than larval parasitoids from Europe and North America [e.g*. Leptopilina heterotoma* Thompson (Hymenoptera, Figitidae)] that are rarely able to complete their development in *D. suzukii* (Chabert et al. 2012; Poyet et al. 2013; Gabarra et al. 2015; Rossi-Stacconi et al. 2015; Daane et al. 2016). In Europe, only the generalist pupal parasitoids such as *Trichopria drosophilae* Perkins (Hymenoptera: Diapriidae) and *Pachycrepoideus vindemmiae* Rondani (Hymenoptera: Pteromalidae) can successfully develop in *D. suzukii* and *T. drosophilae* is considered for augmentative biocontrol, but its efficacy to control *D. suzukii* populations in infested orchards remains to be shown (Knoll et al. 2017; Rossi-Stacconi et al. 2017). Compared to chemical control, classical biological control, when applied properly, is often less harmful to the environment, shows direct benefits for food safety and provides effective long-term control of invasive species. However, potential adverse ecological effects have to be considered prior to the introduction of an exotic biological control agent (Heimpel and Mills 2017). Occasionally, introduced parasitoids and predators have unintended non-target impacts (van Driesche and Hoddle 2016) and, as a consequence, regulatory requirements have become more strict. In most countries, approval for release of classical biological control agents requires risk assessments, including detailed studies on host specificity (Hunt et al. 2008; Mason et al. 2013). Current practices usually include laboratory host specificity tests as a first step to define the fundamental host range of the potential biological control agent (van Driesche and Murray 2004; Bigler et al. 2006; van Lenteren et al. 2006).

Here we present new insights into the host specificity of three larval parasitoids that attack *D. suzukii* in its native range in Asia, *Leptopilina japonica* Novkovic & Kimura*, Ganaspis* cf. *brasiliensis* (Ihering) (Hymenoptera, Figitidae) and *Asobara japonica* Belokobylskij (Hymenoptera, Braconidae), by testing them on a variety of potential European hosts.

## Materials and methods

### Insect rearing

#### Target and non-target species

The starting colony of *D. suzukii* was obtained from fruits of *Rubus* sp. and *Fragaria* sp. collected from various sites in Switzerland in 2015. Around five hundred adult flies were reared in gauze cages (BugDorm-4F4545©) kept at 22 ± 2 °C, 60 ± 10% RH and a photoperiod of 16:8 h (*L*:*D*). Flies were fed with sugar water provided on dental cotton rolls and wet cellulose paper was provided as additional water source. Tubes filled with 10 g of commercial artificial fly diet (Formula 4-24 medium©, Carolina Biological SupplyCo. Burlington, NC), 40 mL of 1.43 g L^−1^ of methyl-4-hydroxylbenzoate (inhibitor of fungal growth) and a pinch of yeast were placed in each cage as a food source and oviposition substrate. Tubes with *D. suzukii* eggs were changed twice a week and placed in incubators at 22 °C, 16 h light until emergence of adults, which were then randomly distributed among the rearing cage.

**Fig. 1:**
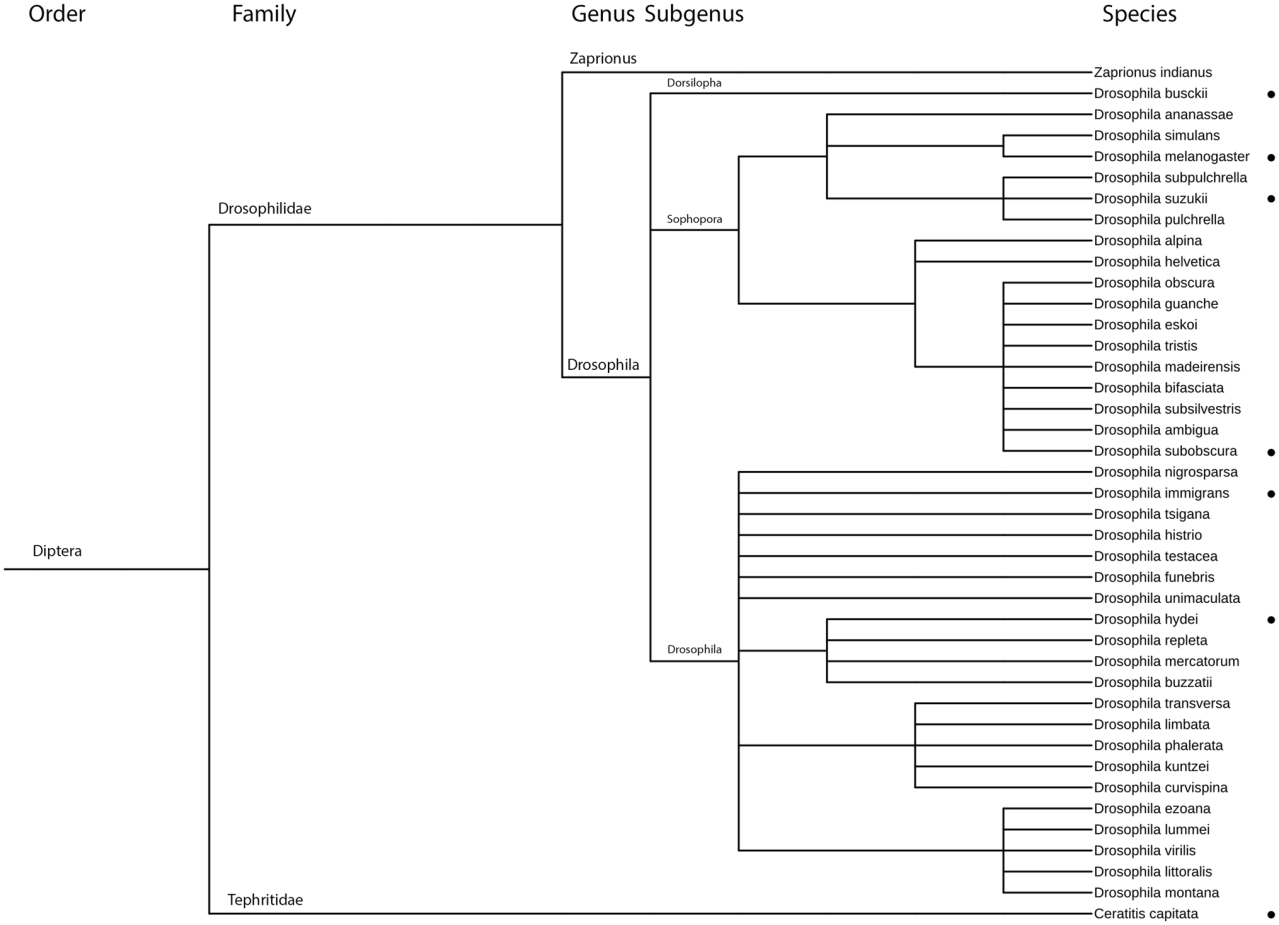
Phylogenetic tree of European *Drosophila* spp. with tested species marked with dots. *Ceratitis capitata* and three exotic Drosophilidae attacking fresh fruits, *Zaprionus indianus*, *Drosophila pulchrella* and *Drosophila subpulchrella*, were added to the tree. The tree is based on Fauna Europaea and the phylogenetic tree was built with NCBI Taxonomy database, Software iTOL (Letunic and Bork 2016)

The selection of non-target hosts was based on phylogenetic relatedness, sympatry of target and non-target species and information on the geographical distribution and occurrence of European Drosophilidae available from the literature (FlyBase database, NCBI Taxonomy database; Kuhlmann et al. 2006; Fleury et al. 2009) (Fig. 1). In total, five European *Drosophila* spp. (*D. busckii* Coquillett, *D. hydei* Sturtevant, *D. immigrans* Sturtevant*, D. melanogaster* Meigen and *D. subobscura* Collin) and one Tephritidae species [*Ceratitis capitata* (Wiedemann)] were selected as non-target test species. The latter was selected as an out-group species as it is also able to oviposit into fresh berries as *D. suzukii,* in contrast to other European *Drosophila* spp. that usually attack decaying fruits and other organic matters. All non-target species were obtained from laboratory colonies of INRA, Sophia-Antipolis, France, in 2015. The *Drosophila* spp. were reared on the same artificial diet and under the same conditions as *D. suzukii*. *Ceratitis capitata* was reared on a homemade artificial diet (10 g of Carolina© artificial fly diet, 10 g of carrot powder and 10 g of yeast powder with 40 mL of 1.43 g L^−1^ of methyl-4-hydroxylbenzoate) in cages of two hundred adult flies (30 × 30 × 30 cm BugDorm-1©).

#### Parasitoid species

The following parasitoids were collected from fruits infested with *D. suzukii* in Asia in 2015 (Girod et al. 2018b) and reared in the quarantine facilities of CABI in Delémont, Switzerland: (1) *Ganaspis* cf. *brasiliensis* from *Prunus cerasoides* D. Don, (Kunming, Yunnan, China) and (2) *Ganaspis* cf. *brasiliensis Prunus serrulata* Lindl. (Tokyo, Japan); (3) *Leptopilina japonica* from *P. cerasoides* (Kunming, Yunnan, China); (4) *Leptopilina japonica* from *Prunus* sp. (Beijing, China); (5) *Asobara japonica* Belokobylskij collected by sweeping over *P. serrulata* fruits (Tokyo, Japan). *Ganaspis brasiliensis*, which has been recorded from various continents (Buffington and Forshage 2016; Nomano et al. 2017), is likely a complex of cryptic species with different distributions and various degrees of specificity (Nomano et al. 2017). Until the taxonomic status of *G. brasiliensis* has been clarified with molecular methods, the two populations from China and Japan used in this study are referred to as *Ganaspis* cf. *brasiliensis*. In addition to the Asian species, we also tested the European *L. heterotoma*, which was collected in Delémont, Switzerland, in summer 2015.

The Asian Figitidae were reared in boxes (ø 90 × 50 mm), each containing ca. 50–60 individuals and kept at 22 ± 2 °C, 60 ± 10% RH and 16 h light. An Eppendorf tube with a wet cellulose paper was added to each rearing box as a water source. Boxes were closed with a foam plug on which a drop of honey was placed as a food source. Fresh blueberries (*Vaccinium corymbosum*) were placed in each *D. suzukii* rearing cage for 48 h for infestation, and then distributed among the parasitoid rearing boxes for another 48 h to allow female parasitoids to oviposit in the young fly larvae. After the exposure infested fruits were removed and kept in rearing tubes (ø 50 × 100 mm) with a filter paper at the bottom to absorb leaking fruit juice. The thelytokous species *A. japonica* and the European *L. heterotoma* were maintained offering the wasps first instar larvae of *D. melanogaster* in artificial diet for 3–4 days. A drop of honey was added to each tube as food source. The rearing tubes were checked daily for newly emerged parasitoids, which were immediately transferred to the rearing boxes.

### Host specificity testing

Before the host specificity tests, the oviposition substrates containing the fly larvae had to be modified due to differences in attractiveness to the parasitoid and fly species. In an earlier study it had been shown that the blended CAROLINA© diet used for rearing the *Drosophila* spp. is not accepted as substrate by *Ganaspis* cf. *brasiliensis* (Girod et al. 2018a). However, besides *D. suzukii,* none of the *Drosophila* species used in this study lays eggs in fresh fruits. Consequently, a blended diet with fruit was developed, which was accepted by all the Drosophilidae, Tephritidae and parasitoids tested (25 g of blended fresh blueberry, 40 mL of 1.43 g L^−1^ of methyl-4-hydroxylbenzoate and 20 g of blended CAROLINA© diet).

Specificity tests were carried out in two steps. In experiment A, *A. japonica, L. japonica* and *L. heterotoma* were tested on *D. suzukii*, *D. melanogaster*, *D. immigrans*, *D. subobscura* and *D. busckii* in plain regular CAROLINA© diet instead of fruit (Table 1, Experiment A), because Girod et al. (2018a) demonstrated that these species would show the same parasitism behaviour with larvae in this artificial diet and blueberries. In experiment B, the two populations of *Ganaspis* cf. *brasiliensis* were tested (1) on the same host species as in experiment A in the modified diet containing blended blueberries, and (2) on *D. suzukii* and *C. capitata* in fresh blueberries (Table 1, Exp. B). Finally, in experiment B, *L. japonica* was also tested on *D. hydei* and *C. capitata* (two species that were not assessed in experiment A). *Asobara japonica* was not assessed further because experiment A had shown that this species is highly polyphagous. In total, 43 different combinations of parasitoids, oviposition substrates and host were tested (Table 1). All experiments were conducted under laboratory conditions at 22 ± 2 °C, 60 ± 10% RH and a photoperiod of 16:8 h (*L*:*D*).

**Table 1:** Experimental testing scheme for each parasitoid, diet, and host (*only tested hosts of *L. japonica* Beijing, China for experiment B)

Experiment #	Parasitoids		Oviposition substrate	Hosts	# Replicates (*n*)
	Species	Origin			
A	*Asobara japonica * *Leptopilina japonica * *Leptopilina japonica * *Leptopilina heterotoma*	Tokyo, Japan Beijing, China Kunming, China Delémont, Switzerland	Artificial diet	*D. melanogaster*	30
				*D. busckii*	30
				*D. subobscura*	30
				*D. immigrans*	30
				*D. suzukii*	30
B	*Ganaspis* cf. *brasiliensis* *Ganaspis* cf. *brasiliensis* Leptopilina japonica*	Kunming, China Tokyo, Japan Beijing, China	Artificial diet + blended blueberry	D. hydei*	30
				*D. melanogaster*	30
				*D. busckii*	60
				*D. subobscura*	30
				*D. immigrans*	30
				D. suzukii*	60
				C. capitata*	30
			Blueberry	D. suzukii*	60
				C. capitata*	30

From the laboratory colonies, 0–12-h-old female parasitoids were collected and placed in tubes with males (sex ratio female/male 2:1) for 72 h to ensure that they were mated and mature at the time of the experiments (Girod et al. 2018a). After 3 days, single female parasitoids were exposed for a period of 48 h to 10–30 *Drosophila* spp. larvae that were 12–20 h old. *Drosophila* spp. show small differences in their development time and exposing the hosts for 48 h ensured that all *Drosophila* spp. were in their first instar and early second instars, the suitable stages for larval parasitoids of *Drosophila* spp. (Carton et al. 1986). Larvae of *C. capitata* were used for experiment after a period of incubation of 24–32 h. At this time larvae had reached the 1st instar and had a similar size as Drosophilidae larvae.

After 2 days of exposure, single female parasitoids were removed. A total of 30 replicates (60 for *D. suzukii* and *D. busckii* in experiment B) and 20 controls (fly larvae without exposure to parasitoids) were performed for each species. Fly and parasitoid emergence was checked daily and all emerging individuals were counted and sexed. The number of flies with encapsulated parasitoid eggs or larvae was recorded under a microscope after squeezing the fly between two microscope glass slides. The few female parasitoids that died during the experiments were excluded from the analysis, as were the tubes without any emergence of flies or parasitoids. Since host attacks by larval parasitoids may result in failed parasitoid offspring development, while causing the host larva to die (see and references Abram et al. 2016 therein), emergence from controls (flies) was compared to emergence from treatments (flies + parasitoids). In an earlier study it was already shown that parasitoids did not lead to a significant increase of larval mortality of *D. suzukii* when hosts were exposed in blueberries (Girod et al. 2018a).

For each specificity test with different parasitoid populations or substrates, a series of parameters was measured: the number of emerged flies (*n*_d_), the number of emerged flies with an encapsulated parasitoid egg (*n*_e_) among the total number of emerged flies (*n*_d_), the number of emerged parasitoids (*n*_p_), the total number of emerged individuals (*n* = *n*_d_ + *n*_p_), and the number of female parasitoids that attacked fly larvae [i.e. wasps for which at least one emerging parasitoid or encapsulated egg was counted (*n*_o_)].

Five parameters of the host-parasitoid interaction were measured for each population and condition:

1. The “Proportion of Ovipositing Females” (POF) corresponds to the number of female parasitoids which had laid at least one egg in fly larvae (*n*_o_) divided by the number of females tested (*N*). It was calculated as ${\text{POF}} = n_{\text{o}} /N$.
2. The “Overall Parasitism Rate” (OPR), which is the proportion of parasitized hosts, i.e. the proportion of flies that contained an encapsulated egg or produced parasitoid offspring. It was calculated as ${\text{OPR}} = \left( {n_{\text{p}} + n_{\text{e}} } \right)/n$ for each parasitoid female.
3. The “Apparent Parasitism Rate” (APR), which is the proportion of parasitoid offspring among the total number of insects that emerged. It was calculated as ${\text{APR}} = n_{\text{p}} /n$ for each parasitoid female.
4. The “Encapsulation Rate” (ER) corresponds to the proportion of emerged flies that contained encapsulated parasitoid eggs or larvae in their abdomens which they had carried over from the parasitized larval stage. It was calculated as ${\text{ER}} = n_{\text{e}} /\left( {n_{\text{p}} + n_{\text{e}} } \right)$ for each parasitoid female.
5. The “Encapsulation Level” (EL), which is estimated as the proportion of emerged flies that contained a capsule among the total number of insects that emerged. EL was calculated as ${\text{EL}} = n_{\text{e}} /n$ for each parasitoid female.

### Statistical analysis

Values of OPR and APR for each species and condition were compared with generalized linear models (Hoffmeister et al. 2006). Since our results were a mixture of zeros and positive values, we used a Tweedie family (Poisson distribution), followed by pairwise comparisons, using the Tukey’s post hoc test. All statistical analyses were performed with the R studio software (version 3.3.3) (R Core Team 2017).

## Results

**Fig. 2:**
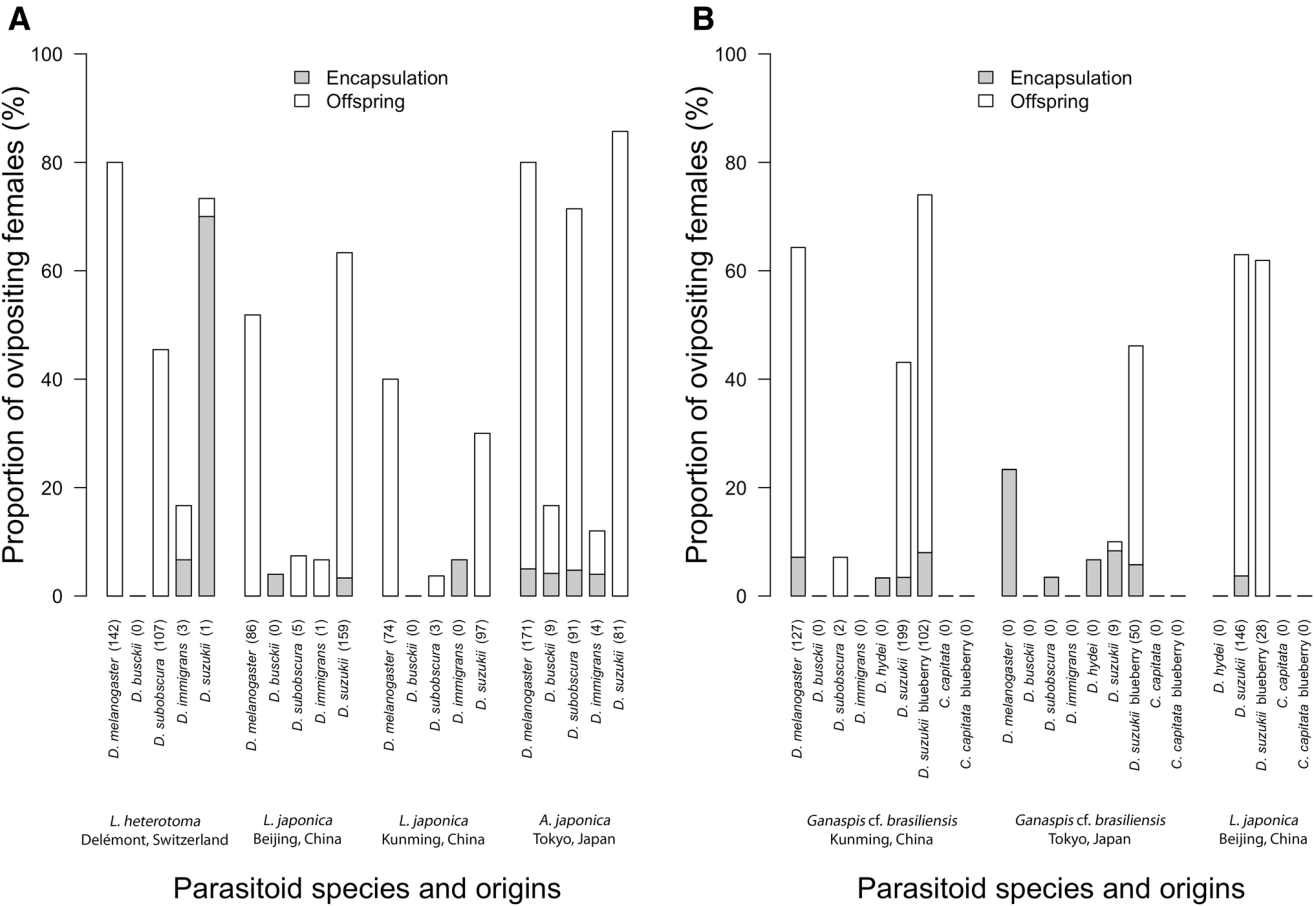
Proportion of ovipositing parasitoid females when exposed to larvae of various hosts in **a** CAROLINA© diet and **b** blueberries or mixed, blended diet. Oviposition was recorded as successful when either an encapsulated egg or larva was found in the abdomen of the emerged fly or when offspring was produced (in brackets, total number of parasitoid offspring)

**Fig. 3:**
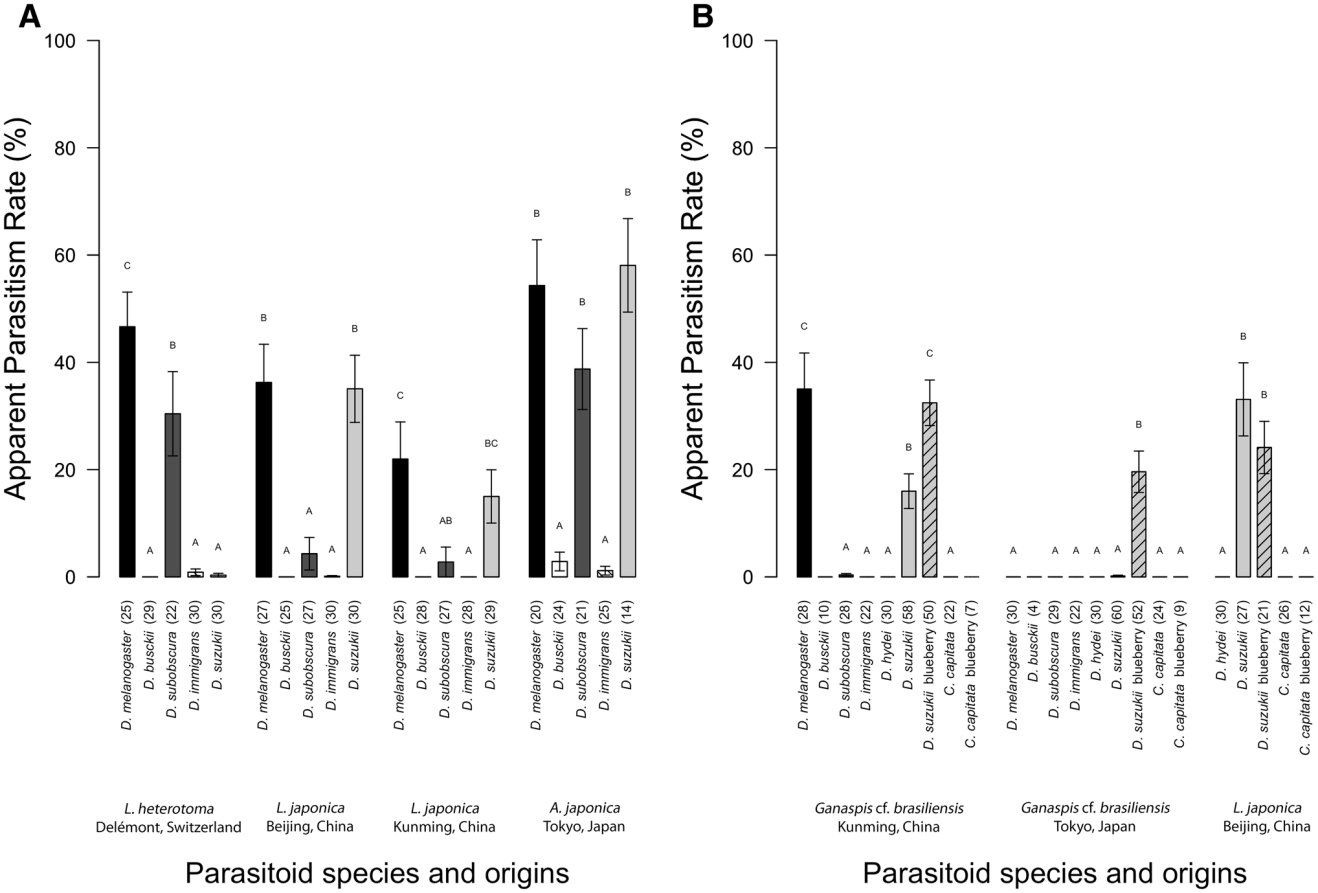
Mean successful parasitism (APR) (± SE) caused by parasitoids exposed to larvae of various hosts in **a** CAROLINA© diet and **b** blueberries or mixed, blended diet. APR was calculated as the proportion of parasitoid emergence among the total number of insects that emerged (in brackets: number of replicates = females included in the calculation). For each parasitoid and experiment, bars with the same letters indicate no significant differences between treatments (GLM (Tweedie family) Tukey post hoc, *p* ≤ 0.05). Tests on *D. busckii* in experiment B were not included in the analyses because of the low number of replicates due to the absence of fly offspring in most tubes

Females of the European parasitoid, *L. heterotoma*, attacked all tested hosts in artificial diet, except *D. busckii*. The proportion of ovipositing females (POF) ranged from 16.7 to 80.0% (Fig. 2a). While this parasitoid managed to produce offspring on *D. melanogaster* and *D. subobscura* with successful parasitism (APR) of 46.7 and 30.4%, respectively, its APR was extremely low (< 1%) on *D. immigrans* and *D. suzukii* (Fig. 3a). Only three females produced offspring on *D. immigrans*. From *D. suzukii* larvae, only a single parasitoid emerged and the encapsulation rate (ER) was as high as 99.4%. In total, around 30% of the emerged flies contained a capsule (EL) in their abdomen (ESM 1). Surprisingly, *L. heterotoma*, was highly attracted to *D. suzukii* with 73.3% of the females laying eggs (POF) in the *D. suzukii* larvae.

The two *L. japonica* populations (Beijing and Kunming) showed similar responses to non-target hosts. The proportions of females from the Beijing population that laid eggs (POF) on *D. melanogaster* and *D. suzukii* were high, i.e. 51.9 and 63.3% respectively, whereas it was 40.0 and 31.0% for the Kunming population. Lower proportions were observed on *D. subobscura* (7.4 and 3.7% for the Beijing population and the Kunming population) and *D. immigrans* (6.7 and 7.1%) (Fig. 2a). Successful parasitism (APR) for the Beijing population on *D. melanogaster* and *D. suzukii* was high, i.e. 36.3 and 35.17% respectively, and 21.9 and 15.0% for the Kunming population. APR was much lower on *D. subobscura* (4.3% for the Beijing population and 2.8% for the Beijing population) and only one progeny emerged from *D. immigrans* (APR = 0.1% in the Beijing population) (Fig. 3a)*. Leptopilina japonica* females did not attack *D. busckii* in artificial diet. *Ceratitis capitata* larvae were parasitized neither in blended diet nor in fresh blueberries. However, on *D. suzukii*, the proportions of females laying eggs on blended diet and blueberries were 63.0 and 61.9%, respectively. In both conditions, successful parasitism was high, reaching 33.1 and 24.1% respectively (Figs. 2b, 3b). Encapsulation levels were low for the two *L. japonica* populations in all parasitized hosts.

*Asobara japonica* females successfully attacked all tested European fly species and *D. suzukii* with a proportion of ovipositing females (POF) ranging from 12.0 to 85.7% (Fig. 2a). Of all tested parasitoids, it showed the highest successful parasitism (APR) on *D. melanogaster* with 54.3%*, D. busckii* with 2.9%, *D. subobscura* with 38.7%, *D. immigrans* with 1.2% and *D. suzukii* with 58.1% in artificial diet (Fig. 3a). *Asobara japonica* eggs and larvae were rarely encapsulated except on *D. immigrans* (ER = 33.3%). Of all parasitoids tested, *A. japonica* was most attracted to *D. suzukii,* with 85.7% of the females laying eggs (POF) in the *D. suzukii* larvae.

Both *Ganaspis* cf. *brasiliensis* populations hardly attacked larvae of D. hydei, *D. immigrans* and *C. capitata,* and no progeny emerged from these hosts (Figs. 2b, 3b). Overall, the proportion of females that oviposited (POF) was higher for the Kunming population (ranging from 3.3 to 74%) than the Tokyo population (ranging from 3.5 to 46.2%) (Fig. 2b). No emergence of parasitoids was observed for the *Ganaspis* cf. *brasiliensis* population from Tokyo on *D. melanogaster* and *D. subobscura,* and the few attempts on the later host (OPR < 1%) were all encapsulated (ER = 100%). In contrast, *Ganaspis* cf. *brasiliensis* from Kunming did manage to develop in high numbers on *D. melanogaster,* and two specimens emerged from *D. subobscura,* with ER 14.8% and 25.0%, respectively. Even on its natural host, i.e. *D. suzukii*, *Ganaspis* cf. *brasiliensis* (Tokyo population) rarely successfully developed on blended diet, with APR as low as 0.2% and ER of 85.0%; however, in blueberry, APR was up to 19.6% and ER was down to 15.6%. The Kunming population performed much better on *D. suzukii* in blended diet, with APR of 15.98% and ER of 18.3%. In blueberry, APR was 32.5% and ER was 15.1% (Fig. 3b).

For none of the parasitoids there were indications that host attacks resulted in increased larval mortality without parasitoid development (ESM2). However, in some cases counting the fly eggs laid in artificial diet accurately was not possible, resulting in higher numbers of emerged individuals than eggs counted at the beginning of the experiment. For these cases, we could not perform a comparison, but the high numbers of emerged individuals suggested no or very low larval mortality.

## Discussion

Among the Asian parasitoids tested, the *Ganaspis* cf. *brasiliensis* population from Tokyo showed the highest degree of host specificity. Successful development was observed exclusively in *D. suzukii* in blueberries. Kasuya et al. (2013a) obtained exactly the same results with a population from the same locality [as ‘*suzukii*-specialized’ type of *Ganaspis xanthopoda* (Kasuya et al. 2013a)]. They carried out laboratory tests and showed that *Ganaspis* cf. *brasiliensis* parasitized *D. suzukii* larvae in fresh cherry fruits, but did not parasitize those in a *Drosophila* artificial diet. In addition, they did not parasitize larvae of the following species: *Drosophila lutescens* Okada, *D. rufa* Kikkawa & Peng, *D. auraria* Peng, *D. biauraria* Block & Wheeler and *D. triauraria* Block & Wheeler even when these occurred in fresh cherry fruits. However, too few replicates were made on these species to draw firm conclusions regarding their suitability as hosts. Surprisingly, in our study, the Kunming population of the supposedly similar parasitoid species showed less specificity, as it very successfully parasitized *D. melanogaster* and *D. suzukii* in the blended diet, whereas in earlier trials, we had failed to rear the same population in an artificial diet without fruits (Girod et al. 2018a). Recent work by Nomano et al. (2017) has shown that the *G. brasiliensis* complex, to which the tested *Ganaspis* cf. *brasiliensis* belongs, includes several cryptic species with totally different host ranges (see also Kasuya et al. 2013b). It is possible that additional cryptic species or biotypes varying in their specificity occur even within the *G. brasiliensis* group that parasitizes *D. suzukii*. Intraspecific variations in host preference or even host specificity are rather common in parasitoids (Höller 1991; Vazquez et al. 2004). It is therefore of upmost importance to improve our understanding of the taxonomy of this group in relation to its specificity before using *Ganaspis* cf. *brasiliensis* in a biological control programme, in order to choose the most suitable and specific population. The same *Ganaspis* cf. *brasiliensis* is the main larval parasitoid of *D. suzukii* in South Korea (Daane et al. 2016), Japan (Kasuya et al. 2013a; Wachi et al. 2015; Matsuura et al. 2017) and China (Girod et al. 2018b), where it probably also attacks two other fruit-inhabiting drosophilids, *D. pulchrella* and *D. subpulchrella*, the two sister species of *D. suzukii*.

In contrast to what was observed for the two *Ganaspis* cf. *brasiliensis* populations, no difference was found between the two populations of *L. japonica* in terms of their degree of specificity. Both populations happily attacked and developed in larvae of *D. melanogaster, D. subobscura* and *D. suzukii* in all substrates but not in the four other hosts, with the exception of one successful development in a *D. immigrans* larva. *Leptopilina japonica* frequently parasitized larvae of *D. suzukii* in Japan (Novković et al. 2011; Matsuura et al. 2017), South Korea (Daane et al. 2016) and China, where it probably attacks also larvae of *D. pulchrella* and *D. subpulchrella* (Girod et al. 2018b). In Japan, it is also found on *D. biauraria* and *D. rufa* and it has been successfully reared on *D. simulans* in the laboratory (Novković et al. 2011). Thus, *L. japonica* is probably not sufficiently specific to be considered for introduction into Europe.

The third Asian parasitoid species tested in this study appeared to be the most polyphagous. *Asobara japonica* attacked all Drosophilidae proposed in the first experiment and was thus excluded from the following tests. This species was already known as a polyphagous parasitoid in Asia, being recorded on more than 25 Drosophilidae species (Nomano et al. 2015). Daane et al. (2016) and Guerrieri et al. (2016) recorded it on *D. suzukii* in South Korea and Mitsui and Kimura (2010), Nomano et al. (2015) and Matsuura et al. (2017) in Japan. Other studies in Europe and North America showed its ability to parasitize *D. suzukii* by affecting the haemocyte load of larvae, thereby overcoming its cellular immune system (Chabert et al. 2012; Kacsoh and Schlenke 2012; Poyet et al. 2013). Despite its abundance, *A. japonica* is rarely obtained from *D. suzukii* in Japanese fresh fruits, possibly because of its attraction for hosts in fermenting fruits, decayed mushrooms and plant leaves (Nomano et al. 2015). However, Biondi et al. (2017) showed that, in the laboratory, *A. japonica* females were able to learn exploiting volatiles emitted by fruits infested by *D. suzukii*. Nevertheless, its polyphagy excludes it from the list of potential candidates for introduction.

The European *L. heterotoma* showed a strong interest in *D. suzukii* larvae; however, the vast majority of parasitoid eggs and larvae were encapsulated by the host, confirming earlier studies (Chabert et al. 2012; Kacsoh and Schlenke 2012; Poyet et al. 2013; Knoll et al. 2017). Only one *L. heterotoma* progeny was able to overcome the immune response of *D. suzukii* and, although in some cases the rate of encapsulation avoidance may be much higher (e.g. 10–30% in Rossi-Stacconi et al. 2015, 2017), parasitism by *L. heterotoma* has not yet been found in the field (Kenis et al. 2016). However, it cannot be ruled out that over time *L. heterotoma* or other native larval parasitoid can become adapted to the new host, as observed in many other cases (Henter and Via 1995; Urbaneja et al. 2000; Jones et al. 2015). Although it was reported in early studies that *D. subobscura* larvae were found to be unable to form a capsule around eggs of the parasitoids *A. tabida* and *L. heterotoma* (Eslin and Doury 2006), capsules were sporadically found in the abdomens of *D. subobscura* that had been parasitized at the larval stage by *Ganaspis* cf. *brasiliensis* (Kunming strain: 1 capsule, Tokyo strain: 2 capsules, ESM 1). Since Eslin and Doury (2006) did not test any *Ganaspis* spp., it remains unclear though whether these rare encapsulations were related to the parasitoid species or the *D. subobscura* strain used in our experiments.

So far, *Ganaspis* cf. *brasiliensis* appears to be the best candidate for introduction into Europe and other invaded regions. It is the main parasitoid of *D. suzukii* in East Asia (Daane et al. 2016; Matsuura et al. 2017; Girod et al. 2018b), and this study showed that it has the narrowest host range. However, our observations that specificity varies with populations implies that further studies are needed to elucidate mechanisms leading to specificity and to investigate the existence of cryptic species or geographic biotypes showing difference in host location, host preference, searching and oviposition behaviour. In addition, more Drosophilidae could be tested with *Ganaspis* cf. *brasiliensis*, however, this study and Girod et al. (2018a) highlighted the difficulty of finding a substrate that is suitable for both *Ganaspis* cf. *brasiliensis* and the non-target species. *Ganaspis* cf. *brasiliensis* prefers ovipositing in fruits, and even a diet with blended fruits appeared not suitable for at least one of the two populations tested. In contrast, most Drosophilidae live in decaying plants and fungal material (van Alphen and Janssen 1981), and will not attack fresh or even rotting fruits and, thus, cannot be tested with *Ganaspis* cf. *brasiliensis*. Besides, showing that a certain biotype of *Ganaspis* cf. *brasiliensis* is specific to larvae in fresh fruits is a strong argument in favour of its probable specificity in regions of introductions where no native Drosophilidae live in fresh fruits. However, prior to releases the taxonomic status of *Ganaspis* cf. *brasiliensis* needs to be clarified and additional tests are needed to proof its efficiency on other fruits and in nature.

## Author contributions

PG, MK and TH conceived research. PG, OL and TU conducted experiments. PG, MK and TH wrote the manuscript. All authors edited the manuscript and approved the final version.

## Electronic supplementary material

Below is the link to the electronic supplementary material.

Supplementary material 1 (PDF 40 kb)

Supplementary material 2 (DOCX 63 kb)
