# Supplementary material for: Host specificity of Asian parasitoids for potential classical biological control of Drosophila suzukii
Source: J Pest Sci (2004). 2018 Jun 18;91(4):1241–50. doi: 10.1007/s10340-018-1003-z (PMC6063322; doi:10.1007/s10340-018-1003-z)
Supplement: Supplementary file 1 — Supplementary material 1 (PDF 40 kb) [file 10340_2018_1003_MOESM1_ESM.pdf]

Table 2. Proportion of ovipositing females, outcomes of oviposition, and measured parameters of the parasitism (success and encapsulation) per parasitoid species, origin, host and substrate.

Parasitoid		Host	Substrate	No. of females tested	Outcomes of ovipositing females (%)			Outcomes of parasitism (%)			
Species	Origin				Proportion of Ovipositing Females (POF)	All encapsulated	At least one offspring	Overall Parasitism Rate (OPR)	Apparent Parasitism Rate (APR)	Encapsulation Level (EL)	Encapsulation Rate (ER)
		D. melanogaster	Artificial diet	30	80.00	0.00	80.00	47.56	46.65	0.92	2.91
		D. busckii	Artificial diet	30	0.00	0.00	0.00	0.00	0.00	0.00	-
Leptopilina heterotoma	Delémont, Switzerland	*D. subobscura*	Artificial diet	30	45.45	0.00	45.45	30.41	30.41	0.00	0.00
		D. immigrans	Artificial diet	30	16.67	6.67	10.00	1.86	0.87	0.99	56.00
		D. suzukii	Artificial diet	30	73.33	70.00	3.33	29.94	0.33	29.60	99.43
		D. melanogaster	Artificial diet	30	51.85	0.00	51.85	36.26	36.26	0.00	0.00
		D. busckii	Artificial diet	30	4.00	4.00	0.00	0.05	0.00	0.05	100.00
Leptopilina japonica	Beijing, China	*D. subobscura*	Artificial diet	30	7.41	0.00	7.41	4.32	4.32	0.00	0.00
		D. immigrans	Artificial diet	30	6.67	3.33	3.33	0.66	0.12	0.54	75.00
		D. suzukii	Artificial diet	30	63.33	3.33	60.00	35.15	35.07	0.08	5.26
		D. melanogaster	Artificial diet	30	40.00	0.00	40.00	21.97	21.97	0.00	0.00
		D. busckii	Artificial diet	30	0.00	0.00	0.00	0.00	0.00	0.00	-
Leptopilina japonica	Kunming, China	*D. subobscura*	Artificial diet	30	3.70	0.00	3.70	2.78	2.78	0.00	0.00
		D. immigrans	Artificial diet	30	7.14	7.14	0.00	0.26	0.00	0.26	100.00
		D. suzukii	Artificial diet	30	31.03	0.00	31.03	15.00	15.00	0.00	0.00
		D. melanogaster	Artificial diet	30	80.00	5.00	75.00	54.94	54.31	0.63	6.25
		D. busckii	Artificial diet	30	16.67	4.17	12.50	3.12	2.87	0.25	25.00
Asobara japonica	Tokyo, Japan	*D. subobscura*	Artificial diet	30	71.43	4.76	66.67	38.85	38.74	0.11	6.67
		D. immigrans	Artificial diet	30	12.00	4.00	8.00	2.26	1.17	1.09	33.33
		D. suzukii	Artificial diet	30	85.71	0.00	85.71	59.27	58.08	1.19	2.08
		D. melanogaster	Blended diet	30	64.29	7.14	57.14	37.49	35.02	2.47	14.78
		D. busckii	Blended diet	60	0.00	0.00	0.00	0.00	0.00	0.00	-
		D. subobscura	Blended diet	30	7.14	0.00	7.14	0.47	0.36	0.11	25.00
		D. immigrans	Blended diet	30	0.00	0.00	0.00	0.00	0.00	0.00	-
Ganaspis cf. *brasiliensis*	Kunming, China	*D. hydei*	Blended diet	30	3.33	3.33	0.00	0.10	0.00	0.10	100.00
		D. suzukii	Blended diet	60	43.10	3.45	39.66	18.61	15.98	2.63	18.34
		D. suzukii	Blueberry	60	74.00	8.00	66.00	36.95	32.46	4.49	15.09
		C. capitata	Blended diet	30	0.00	0.00	0.00	0.00	0.00	0.00	-
		C. capitata	Blueberry	30	0.00	0.00	0.00	0.00	0.00	0.00	-
		D. melanogaster	Blended diet	30	23.33	23.33	0.00	0.96	0.00	0.96	100.00
		D. busckii	Blended diet	60	0.00	0.00	0.00	0.00	0.00	0.00	-
		D. subobscura	Blended diet	30	3.45	3.45	0.00	0.43	0.00	0.43	100.00
		D. immigrans	Blended diet	30	0.00	0.00	0.00	0.00	0.00	0.00	-
Ganaspis cf. *brasiliensis*	Tokyo, Japan	*D. hydei*	Blended diet	30	6.67	6.67	0.00	0.61	0.00	0.61	100.00
		D. suzukii	Blended diet	60	10.00	8.33	1.67	0.48	0.16	0.32	85.00
		D. suzukii	Blueberry	60	46.15	5.77	40.38	21.20	19.60	1.60	15.63
		C. capitata	Blended diet	30	0.00	0.00	0.00	0.00	0.00	0.00	-
		C. capitata	Blueberry	30	0.00	0.00	0.00	0.00	0.00	0.00	-
		D. hydei	Blended diet	30	0.00	0.00	0.00	0.00	0.00	0.00	-
		D. suzukii	Blended diet	30	62.96	3.70	59.26	33.29	33.10	0.19	5.88
Leptopilina japonica	Beijing, China	*D. suzukii*	Blueberry	30	61.90	0.00	61.90	24.13	24.13	0.00	0.00
		C. capitata	Blended diet	30	0.00	0.00	0.00	0.00	0.00	0.00	-
		C. capitata	Blueberry	30	0.00	0.00	0.00	0.00	0.00	0.00	-
