# Supplementary material for: Host specificity of Asian parasitoids for potential classical biological control of Drosophila suzukii
Source: J Pest Sci (2004). 2018 Jun 18;91(4):1241–50. doi: 10.1007/s10340-018-1003-z (PMC6063322; doi:10.1007/s10340-018-1003-z)
Supplement: Supplementary file 2 — Supplementary material 2 (DOCX 63 kb) [file 10340_2018_1003_MOESM2_ESM.docx]

**ESM 2**. Mean number of fly eggs, emerged flies and parasitoids and unknown mortality measured per vial according to parasitoid and fly species and food type tested. (* overall emergence was higher than number of eggs counted, ** for *C. capitata* it was not possible to count eggs, as many were laid in the same oviposition hole, *** due to no emergence in controls host specificity tests with *D. busckii* on blended blueberry diet were excluded from the analysis). Statistical analyses *P*: pairwise comparison of unknown mortality in rearing controls and test conditions (GLM (Tweedie family) Tukey post hoc, p ≤ 0.05).

| Parasitoid species | Fly species | Food type | N (# replicates) | Mean (SE) number of eggs per vial | Mean (SE) number of flies per vial | Mean (SE) number of parasitoids per vial | Mean (SE) number of unknown mortality per vial | Experiment # | *P* |
| --- | --- | --- | --- | --- | --- | --- | --- | --- | --- |
| *Leptopilina heterotoma* JURA | *D. busckii* | Artificial diet | 30 | 17 (1.25) | 13.66 (2.14) | 0 | 24.45 (9.38) | A | ns. |
| *Leptopilina heterotoma* JURA | *D. melanogaster* | Artificial diet | 30 | 17.44 (1.29) | 6.32 (1.1) | 5.68 (0.95) | 30.78 (4.84) | A | ns. |
| *Leptopilina heterotoma* JURA | *D. subobscura* | Artificial diet | 30 | 18.68 (1.32) | 10.32 (2.51) | 4.86 (1.42) | 22.23 (9.51) | A | ns. |
| *Leptopilina heterotoma* JURA | *D. immigrans* | Artificial diet | 30 | 17.23 (1.30) | 14.57 (2.7) | 0.1 (0.06) | 14.12 (10.39) | A | ns. |
| *Leptopilina heterotoma* JURA | *D. suzukii* | Artificial diet | 30 | 16.2 (1.04) | 15.1 (1.43) | 0.03 (0.03) | 5.5 (8.64) | A | ns. |
| *Leptopilina japonica* BEIJING | *D. busckii* | Artificial diet | 30 | 17.76 (1.37) | 15.96 (2.96) | 0 | 0.17 (21.94) | A | ns. |
| *Leptopilina japonica* BEIJING | *D. melanogaster* | Artificial diet | 30 | 16.48 (1.23) | 5.52 (1.08) | 3.19 (0.74) | 45.58 (5.1) | A | ns. |
| *Leptopilina japonica* BEIJING | *D. subobscura* | Artificial diet | 30 | 16.63 (1.18) | 9.59 (1.52) | 0.19 (0.15) | 37.62 (10.73) | A | ns. |
| *Leptopilina japonica* BEIJING | *D. immigrans* | Artificial diet | 30 | 16.77 (1.27) | 10.47 (1.33) | 0.03 (0.03) | 32.39 (9.37) | A | ns. |
| *Leptopilina japonica* BEIJING | *D. hydei* | Blended blueberry | 30 | 21.6 (1.37) | 15.23 (1.2) | 0 | 27.73 (4.06) | B | ns. |
| *Leptopilina japonica* BEIJING | *D. suzukii* | Artificial diet | 30 | 18.9 (1.2) | 10.8 (1.7) | 5.3 (1.07) | 11.51 (6.09) | A | ns. |
| *Leptopilina japonica* BEIJING | *D. suzukii* | Blended blueberry | 30 | 17.33 (1.08) | 11 (1.53) | 5.41 (1.37) | 3.75 (6.68) | B | - |
| *Leptopilina japonica* BEIJING | *D. suzukii* | Blueberry | 30 | 17.38 (1.34) | 4.43 (0.87) | 1.33 (0.32) | 63.64 (5.02) | B | ns. |
| *Leptopilina japonica* BEIJING | *C. capitata* | Blended blueberry | 30 | 17.88 (0.86) | 4.65 (0.39) | 0 | 73.04 (2.67) | B | ns. |
| *Leptopilina japonica* BEIJING | *C. capitata* | Blueberry | 30 | - | 2.08 (0.4) | 0 | -** | B | - |
| *Leptopilina japonica* KUNMING | *D. busckii* | Artificial diet | 30 | 16.96 (1.04) | 18.21 (3.37) | 0 | 0* | A | - |
| *Leptopilina japonica* KUNMING | *D. melanogaster* | Artificial diet | 30 | 17.72 (1.39) | 11.6 (1.54) | 2.96 (1) | 16.82 (5.57) | A | ns. |
| *Leptopilina japonica* KUNMING | *D. subobscura* | Artificial diet | 30 | 20.63 (1.26) | 16.63 (2.33) | 0.11 (0.11) | 17.52 (13.06) | A | ns. |
| *Leptopilina japonica* KUNMING | *D. immigrans* | Artificial diet | 30 | 16.43 (1.2) | 14.5 (1.17) | 0 | 6.64 (6.39) | A | ns. |
| *Leptopilina japonica* KUNMING | *D. suzukii* | Artificial diet | 30 | 16.03 (1.08) | 13.41 (1.35) | 3.34 (1.23) | 0* | A | - |
| *Asobara japonica* TOKYO | *D. busckii* | Artificial diet | 30 | 17.71 (1.23) | 12.46 (1.76) | 0.38 (0.23) | 26.78 (9.92) | A | ns. |
| *Asobara japonica* TOKYO | *D. melanogaster* | Artificial diet | 30 | 18.15 (1.52) | 5.1 (1.1) | 8.55 (1.81) | 26.04 (5.49) | A | ns. |
| *Asobara japonica* TOKYO | *D. subobscura* | Artificial diet | 30 | 18.05 (1.52) | 8.19 (2.16) | 4.33 (0.98) | 32.18 (10.64) | A | ns. |
| *Asobara japonica* TOKYO | *D. immigrans* | Artificial diet | 30 | 16.56 (1.37) | 10.84 (1.3) | 0.16 (0.11) | 27.83 (8.68) | A | ns. |
| *Asobara japonica* TOKYO | *D. suzukii* | Artificial diet | 30 | 19 (1.76) | 4.79 (2.18) | 5.79 (1.28) | 45.74 (7.55) | A | ns. |
| *Ganaspis cf. brasiliensis* KUNMING | *D. hydei* | Blended blueberry | 30 | 20.37 (1.42) | 14.77 (1.50) | 0 | 28.67 (4.86) | B | ns. |
| *Ganaspis cf. brasiliensis* KUNMING | *D. melanogaster* | Blended blueberry | 30 | 21.07 (1.04) | 11.96 (2.04) | 4.53 (1.04) | 22.94 (6.42) | B | ns. |
| *Ganaspis cf. brasiliensis* KUNMING | *D. subobscura* | Blended blueberry | 30 | 20.18 (1.14) | 20.54 (2.33) | 0.07 (0.05) | 0* | B | - |
| *Ganaspis cf. brasiliensis* KUNMING | *D. immigrans* | Blended blueberry | 30 | 20.86 (1.08) | 14.27 (2.37) | 0 | 29.93 (9.99) | B | ns. |
| *Ganaspis cf. brasiliensis* KUNMING | *D. suzukii* | Blended blueberry | 30 | 20 (0.86) | 26.95 (3.07) | 3.43 (0.77) | 0* | B | - |
| *Ganaspis cf. brasiliensis* KUNMING | *D. suzukii* | Blueberry | 60 | 16.28 (0.75) | 4.18 (0.37) | 2.04 (0.32) | 58.83 (2.86) | B | ns. |
| *Ganaspis cf. brasiliensis* KUNMING | *C. capitata* | Blended blueberry | 60 | 17.45 (1.14) | 5.64 (1.03) | 0 | 65.8 (5.39) | B | ns. |
| *Ganaspis cf. brasiliensis* KUNMING | *C. capitata* | Blueberry | 30 | - | 1 | 0 | -** | B | - |
| *Ganaspis cf. brasiliensis* TOKYO | *D. hydei* | Blended blueberry | 30 | 19.27 (1.08) | 15.2 (1.2) | 0 | 21.51 (4.33) | B | ns. |
| *Ganaspis cf. brasiliensis* TOKYO | *D. melanogaster* | Blended blueberry | 30 | 22.73 (1.26) | 24.3 (2.24) | 0 | 0* | B | - |
| *Ganaspis cf. brasiliensis* TOKYO | *D. subobscura* | Blended blueberry | 30 | 22.97 (1.36) | 22.62 (3.26) | 0 | 5.18 (10.51) | B | - |
| *Ganaspis cf. brasiliensis* TOKYO | *D. immigrans* | Blended blueberry | 30 | 21.36 (1.17) | 12 (1.52) | 0 | 40.95 (6.95) | B | ns. |
| *Ganaspis cf. brasiliensis* TOKYO | *D. suzukii* | Blended blueberry | 60 | 19.63 (0.78) | 26.3 (2.21) | 0.15 (0.15) | 0* | B | - |
| *Ganaspis cf. brasiliensis* TOKYO | *D. suzukii* | Blueberry | 60 | 14.17 (0.81) | 4 (0.41) | 0.96 (0.2) | 64.26 (2.9) | B | ns. |
| *Ganaspis cf. brasiliensis* TOKYO | *C. capitata* | Blended blueberry | 30 | 15.67 (0.75) | 4.92 (0.54) | 0 | 68.97 (2.96) | B | ns. |
| *Ganaspis cf. brasiliensis* TOKYO | *C. capitata* | Blueberry | 30 | - | 2.33 (0.5) | 0 | -** | B | - |
| Rearing control | *D. hydei* | Blended blueberry | 20 | 20.6 (1.23) | 15.25 (1.46) | - | 26.8 (5.22) |  |  |
| Rearing control | *D. melanogaster* | Artificial diet | 20 | 18.9 (1.36) | 12.85 (1.50) | - | 33.1 (6.05) |  |  |
| Rearing control | *D. melanogaster* | Blended blueberry | 20 | 23.15 (1.58) | 22.3 (2.42) | - | 6.22 (6.76) |  |  |
| Rearing control | *D. busckii* | Artificial diet | 20 | 17.15 (1.45) | 10.55 (2.06) | - | 28.87 (13.83) |  |  |
| Rearing control | *D. busckii* | Blended blueberry | 20 | 21.2 (1.53) | 0 | - | 100 (0)*** |  |  |
| Rearing control | *D. subobscura* | Artificial diet | 20 | 21.05 (1.59) | 13.3 (1.88) | - | 34.18 (9.34) |  |  |
| Rearing control | *D. subobscura* | Blended blueberry | 20 | 17.5 (1.45) | 22.4 (4.47) | - | 0* |  |  |
| Rearing control | *D. immigrans* | Artificial diet | 20 | 17.25 (1.47) | 11.15 (2.14) | - | 26.19 (12.91) |  |  |
| Rearing control | *D. immigrans* | Blended blueberry | 20 | 24.75 (1.31) | 7.3 (1.72) | - | 32.02 (8.57) |  |  |
| Rearing control | *D. suzukii* | Artificial diet | 20 | 15.2 (1.36) | 13.05 (2.06) | - | 15.9 (11.23) |  |  |
| Rearing control | *D. suzukii* | Blended blueberry | 20 | 17.5 (1.45) | 20.5 (2.84) | - | 0* |  |  |
| Rearing control | *D. suzukii* | Blueberry | 40 | 16.93 (1.15) | 4.7 (0.63) | - | 69.64 (4.21) |  |  |
| Rearing control | *C. capitata* | Blended blueberry | 20 | 18.5 (1.11) | 5.3 (0.71) | - | 72.36 (3.02) |  |  |
| Rearing control | *C. capitata* | Blueberry | 20 | - | 0.8 (0.3) | - | -** |  |  |
